# Effect of Alpha2-Plasmin Inhibitor C-Terminal Heterogeneity on Clot Lysis and Clot Structure

**DOI:** 10.3390/biom15081127

**Published:** 2025-08-05

**Authors:** Réka Bogáti, Barbara Baráth, Dóra Pituk, Rita Orbán-Kálmándi, Péter Szűcs, Zoltán Hegyi, Zsuzsanna Bereczky, Zsuzsa Bagoly, Éva Katona

**Affiliations:** 1 Division of Clinical Laboratory Science, Department of Laboratory Medicine, Faculty of Medicine, University of Debrecen, 4032 Debrecen, Hungary; bogati.reka@med.unideb.hu (R.B.); pituk.dora@med.unideb.hu (D.P.); zsbereczky@med.unideb.hu (Z.B.); bagoly@med.unideb.hu (Z.B.); 2 Kálmán Laki Doctoral School, University of Debrecen, 4032 Debrecen, Hungary; 3 Department of Biochemistry, Semmelweis University, 1094 Budapest, Hungary; barath.barbara@semmelweis.hu; 4 Healthcare Industry Institute, Faculty of Pharmacy, University of Debrecen, 4032 Debrecen, Hungary; kalmandi.rita@med.unideb.hu; 5 Department of Anatomy, Histology and Embryology, Faculty of Medicine, University of Debrecen, 4032 Debrecen, Hungary; szucs.peter@med.unideb.hu (P.S.); hegyiz@anat.med.unideb.hu (Z.H.); 6 MTA-DE Lendület “Momentum” Hemostasis and Stroke Research Group, 4032 Debrecen, Hungary

**Keywords:** alpha2-plasmin inhibitor, antiplasmin, fibrinolysis, clot lysis, confocal laser scanning microscopy

## Abstract

Alpha2-plasmin inhibitor (α2PI) has a heterogeneous structure due to proteolytic cleavages in the circulation. The C-terminally cleaved form loses the plasminogen binding site and is, therefore, a slow plasmin inhibitor (NPB-α2PI). As FXIII primarily crosslinks the plasminogen-binding intact form (PB-α2PI) to fibrin, the effect of NPB-α2PI on fibrinolysis has been less studied. Herein, we investigated the effect of C-terminal truncation. Total-, PB-, and NPB-α2PI antigen levels and α2PI incorporation were measured by ELISAs from samples of 80 healthy individuals. Clot lysis parameters of the same subjects were investigated using an in vitro clot lysis assay. α2PI incorporation into the clot was demonstrated by Western blotting. Clot lysis and clot structure were also analyzed using an α2PI-deficient plasma substituted with recombinant PB- and NPB-α2PI. Both plasma and clot-bound levels of total- and NPB-α2PI showed a significant positive correlation with clot lysis parameters. NPB-α2PI was detected in the clot due to non-covalent binding. Regardless of the type of binding, both forms affected the clot structure by increasing the thickness of the fibrin fibers and reducing the pore size. In conclusion, we found that NPB-α2PI can bind non-covalently to fibrin, and this binding contributes to changes in clot structure and inhibition of fibrinolysis.

## 1. Introduction

Alpha2-plasmin inhibitor (α2PI, also named as α2-antiplasmin), the major physiological inhibitor of plasmin, has an important role in the fibrinolytic system by controlling plasmin activity [1]. Congenital deficiency of α2PI leads to severe hemorrhagic disorder due to increased predisposition to fibrinolysis [2]. Elevated fibrinogen and reduced fibrinolysis were demonstrated as risk factors for venous and arterial thrombotic events in clinical studies [3,4,5,6,7].

α2PI is a ≈67 kDa single-chain glycoprotein consisting of 464 amino acids, primarily synthesized by the liver. The human plasma concentration was estimated to be 1 µM [8]. It belongs to the serine protease inhibitor family (serpin) and has a dual function in plasmin inhibition. By forming an irreversible, plasmin–α2–antiplasmin complex (PAP-complex), the function of plasmin is inhibited, and therefore, the dissolution of the fibrin clot becomes slower. This process goes in two steps. First, the C-terminus of α2PI reversibly interacts with the plasmin(ogen) Lys-binding sites. In the second step, a covalent bond is formed between the reactive site of α2PI and the active site of plasmin [9]. Another method of clot stabilization is achieved by FXIII-mediated covalent binding of α2PI to fibrin Aα chains in the formation of fibrin mesh [10]. This cross-linking is responsible for the antifibrinolytic effect of FXIII, not the cross-linking of γ-chains [11]. It has been shown that α2PI can also bind to fibrin noncovalently, which may contribute to the formation of crosslinking by the proper orientation of the crosslinking sites [12].

Alpha2-plasmin inhibitor in the plasma undergoes proteolytic cleavages, resulting in four plasmatic forms. The full-length form (Met-α2PI) is truncated on the N-terminus between Pro12 and Asn13 amino acids by the fibroblast activation protein (sFAP, also called antiplasmin cleaving enzyme (APCE)), resulting in a 452 amino acid variant (Asn-α2PI) [13,14], which accounts for approximately 70% of α2PI in the normal human plasma [15]. Asn-α2PI is a better substrate for the active FXIII. Thus, it is cross-linked 13 times faster to fibrin than the full-length form (Met1-α2PI) [13]. In contrast to other serpins, α2PI has a C-terminal extension, which contains a number of Lys residues and binds to Lys-binding kringles of plasmin(ogen), increasing the inhibitory efficiency of α2PI [16,17]. In the circulation, α2PI could also be modified on the C-terminus. The C-terminally intact form can bind with plasmin(ogen) (PB-α2PI, plasminogen binding variant), while the truncated form loses the plasminogen binding site (NPB-α2PI, non-plasminogen binding variant). NPB-α2PI is a much less potent inhibitor of plasmin, and the binding of α2PI to fibrin by activated FXIII primarily involves the PB form [18,19,20]. A C-terminal cleavage site was demonstrated at Gln421-Asp422, but other potential cleavage sites were also suggested. The protease(s) responsible for C-terminal modification have not yet been determined. The amount of NPB-α2PI in the plasma of healthy controls was reported to be ≈35% [21]. There is a limited amount of information available on the C-terminal proteolytic variations of α2PI in different pathological conditions. A decreased level of full-length and unaltered C-terminally truncated percentage of α2PI was reported in male myocardial infarction survivors at least two months after the acute event [22]. In our previous study, total-α2PI levels proved to be elevated in patients with venous thromboembolism, and the elevation is caused by the elevation of NPB-α2PI levels with no significant change in PB-α2PI levels, compared to healthy controls. Elevated NPB-α2PI levels are independently associated with VTE risk (OR: 9.868; CI: 4.095- 23.783) [5].

During blood clot formation, several proteins, apart from α2PI, can bind to fibrin and affect the structure of the fibrin network, such as actin, albumin, α1-antitrypsin, carboxypeptidase N, coagulation factor XIII, lipoprotein(a), fibronectin, plasminogen, plasminogen activator inhibitor-1, thrombin-activatable fibrinolysis inhibitor (TAFI), thrombospondin, and tissue-type plasminogen activator (t-PA) [23], and the final network structure is highly responsive to the presence of these proteins [24].

The present study aimed to investigate the consequence of C-terminal truncation on the incorporation of α2PI into the fibrin clot and its association with clot lysis and clot structure. We measured total, PB-, and NPB-α2PI levels in the plasmas of healthy individuals and in clots prepared from the same plasmas. The correlation between the measured α2PI levels and the lysis time of the clots prepared from the plasmas was also examined. The clot lysis and clot structure were also analyzed using an α2PI-deficient plasma substituted with recombinant PB- and NPB-α2PI.

## 2. Materials and Methods

### 2.1. Materials

Total-α2PI from human plasma was purified by affinity chromatography using the same monoclonal anti-α2PI antibody that was used in the total-α2PI ELISA. PB-α2PI was extracted from the total α2PI preparation by plasminogen sepharose affinity chromatography to obtain NPB-α2PI. Recombinant proteins were purchased from Sino Biological (Beijing, China): the full-length form of Human Serpin F2 Protein (Full-length α2PI (PB-α2PI)1-464 aa) and the C terminally truncated Human Serpin F2 Protein (1-437 aa). Alpha-2-Antiplasmin Deficient Plasma was purchased from Affinity Biologicals (Affinity Biologicals, ON, Canada). Thrombin from human plasma was purchased from Sigma-Aldrich (St. Louis, MO, USA). Innovin (Siemens, Marburg, Germany) was used as a human tissue factor, and Actilyse (Boehringer Ingelheim International, Germany) as rt-PA. For measuring clot lysis time, samples were added to Greiner 96-well half-area microtiter plates (Greiner Bio-one, Kremsmünster, Austria). Absorbance was measured by a Tecan Infinite 200 spectrophotometer (TECAN Trading AG, Männedorf, Switzerland). Investigation of clot structure was performed in an Ibidi µ-Slide VI slide (ibidi GmbH, Gräfelfing, Germany). Fibrinogen from human plasma (Sigma-Aldrich, St. Louis, MO, USA) was labeled with an Alexa-Fluor 647 (AF647) (Life Technologies, Waltham, MA, USA) in our laboratory.

### 2.2. Methods

#### 2.2.1. Samples

Eighty plasma samples from healthy individuals were randomly selected from the sample pool of our previous study on the effect of α2-plasmin inhibitor heterogeneity on the risk of venous thromboembolism [5]. All chronic diseases except for moderate hypertension (blood pressure between 145/90 and 165/95 mmHg) and any acute illness in the previous 3 weeks were considered as exclusion criteria for healthy controls. Blood was taken from the antecubital vein into vacutainer tubes (Beckton Dickinson, Franklin Lakes, NJ, USA) containing 1/10 volume of 0.109 M citrate between 8 am and 11 am. Plasma was separated by centrifugation at 1500× *g* for 20 min, and aliquots were stored at −70 °C until measurements. All enrolled individuals gave written informed consent. The study fully complied with the Declaration of Helsinki. Ethical approval was obtained from the Regional Ethics Committee at the University of Debrecen, Hungary (ETT TUKEB: 54005-3/2016/EKU).

#### 2.2.2. α2PI Measurements

The total-α2PI antigen concentration was measured by an in-house sandwich ELISA, as previously described [25]. This assay measures all plasmatic forms of α2PI and is not influenced by the presence of plasmin–antiplasmin complexes (reference range of plasma α2PI: 48–85 mg/L). PB-α2PI concentration was measured by an in-house sandwich ELISA, as previously described [5]. NPB-α2PI was calculated by subtraction of PB-α2PI from the total-α2PI.

#### 2.2.3. Investigation of α2PI Incorporation into the Plasma Clot

Healthy plasma samples (n = 80) were clotted by adding 2 U/mL thrombin and 20 mM CaCl_2_. After incubation at 37 °C for 30 min, serum samples, derived from the extrusion of fluid after plasma clotting, were separated by centrifugation (16,100× *g*, 5 min). Total-, PB-, and NPB-α2PI antigen levels were measured from the original plasma samples and from the obtained serum samples using the ELISA assays as described above. Values measured in the serum were multiplied by the dilution factor (1.11) caused by the addition of the coagulation activation mix. Incorporated α2PI forms were calculated by subtracting the amount of α2PI measured in the serum from the corresponding value measured in the plasma.

α2PI incorporation into the clot was also investigated by Western blot. Three randomly selected samples were clotted as above in the presence or absence of 2 mM iodoacetamide (IAA). After intensive washing with 20 × 500 μL PBS, pH 7.2, containing 3 mg/mL IAA, clots were dissolved in Laemmli buffer containing 5% mercaptoethanol and 8 M urea at room temperature for 20 h and analyzed by SDS-PAGE on 7.5% polyacrylamide gels. Proteins transferred to the PVDF membrane (Bio-Rad, Hercules, CA, USA) were immunostained with horseradish peroxidase (HRP)-labeled polyclonal anti-α2PI antibody (GA2AP-AP, Affinity Biologicals, ON, Canada) or monoclonal anti-PB-α2PI antibody (Monoclonal-anti-α2AP 3AP antibody, Technoclone, Vienna, Austria), followed with anti-mouse IgG-HRPO (Southern Biotech, Birmingham, AL, USA) and ECL chemiluminescent reagent (Thermo Fisher Scientific, Waltham, MA, USA) was used for detection.

#### 2.2.4. Clot Lysis Assays (CLA)

An in vitro clot lysis assay was performed on 80 normal plasma samples, as previously described [26]. The time needed to reach 50% clot lysis (CLT50) was defined as the time from the midpoint of the clear-to-maximum-turbid transition, which represents clot formation, to the midpoint of the maximum-turbid-to-clear transition, representing the clot lysis.

To investigate the effect of recombinant full-length and C-terminally truncated α2PI on clot lysis, a modified clot lysis assay was performed. Alpha-2-antiplasmin-deficient plasma was substituted with different recombinant α2PI proteins. The activation mix for clot formation contained 1 U/mL thrombin and 1 mM CaCl_2_ in HEPES buffer (10 mM HEPES, 150 mM NaCl, 0.05% Tween 20, pH 7.4). Plasma mix contained 1.3 times diluted plasma, 100 × diluted Innovin, and 130 ng/mL rt-PA (in HEPES buffer). 50 µL/well plasma mix and 25 µL/well activator mix were pipetted into 96-well microtiter plates. Absorbance was measured by a Tecan Infinite 200 spectrophotometer immediately after mixing at 340 nm, every 1 min, for 72 min at 37 °C. Shiny App software (Simple clot lysis analysis app version 0.3.1) was used to fit the turbidimetry curves of fibrin formation and lysis and determine different parameters of the curve, such as the maximum clot amplitude/absorbance (MaxAbs), 50% clot lysis time (CLT50, time from 50% maximum absorbance to 50% lysis), and area under the curve (AUC).

#### 2.2.5. Clot Structure Analysis with Confocal Laser Scanning Microscopy

Alpha-2-antiplasmin-deficient plasma supplemented with different recombinant α2PI forms was also substituted with AF647-labeled fibrinogen to obtain a 2% labeled fibrinogen fraction. Clot formation was induced by adding 50 µL activation mix containing 0.5 U/mL thrombin and 10 mM CaCl_2_ in TRIS/HCl buffer (0.05M TRIS, 0.1 M NaCl, pH 7.5) to 50 µL plasma. Forty µL samples were loaded immediately into channels of an Ibidi µ-Slide VI and incubated in the dark for two hours at room temperature in a wet chamber. Wells on both sides of the channel were filled with TRIS/HCl buffer. On one side, a 2.5 mL syringe was plugged into the well and filled up with TRIS/HCl buffer to 2 mL. Clots were washed for 2 h and investigated with an Olympus FluoView 3000 confocal microscope (Olympus, Tokyo, Japan, objective lens (UPLFLN 40× dry (refractive index = 1.0, working distance = 0.51 mm, numerical aperture (NA) was 0.75 and pinhole was 280 µm))). The total thickness of the Z-stack measured 42 µm, with a Z-stack size of 2.0 µm, resulting in a total of 22 Z-slices. For confocal image analysis of the clot structures, an open-source software, Fiji (version 2.3) (Fiji Is Just ImageJ), was used. To determine the pore size of the clots, the average radius of bubbles that can fit into the 2D pores and produce maximum coverage of the entire 2D image was calculated; the procedure employed was based on the source code developed by Münster et al. [27]. The fiber width was calculated using a MATLAB (R2019a) GUI called CT-FIRE v3.0 beta (Curvelet Transform—Fiber Extraction) [28]. Twenty-two images (1024 × 1024 pixels) of each slide were recorded in the same positions in a Z-stack. Different parameters (% area covered, pore-size (average radius of bubbles), fiber width) were calculated from the 22 evaluated images and expressed as mean ± SD [29,30].

#### 2.2.6. Other Laboratory Methods

FXIII activity was measured by an ammonia release assay [31] using the Technochrom FXIII chromogenic assay (Technoclone, Vienna, Austria). FXIII-A_2_B_2_ antigen was determined by sandwich ELISA, as described earlier [32]. Fibrinogen concentration was measured using the Clauss method. Plasminogen was measured using the Berichrom Plasminogen assay (Siemens Healthcare Diagnostics GmbH, Marburg, Germany) on the BCS XP Coagulation System.

### 2.3. Statistical Methods

A correlation sample size calculator was used to calculate the required sample size. The required sample size is N = 80 to establish a statistically significant correlation of r > 0.300 at alpha = 0.05 and beta = 0.2. The Kolmogorov–Smirnov test was used for the distribution of the data. Data are presented as mean ± SD or median (interquartile range) depending on the distribution. To investigate the correlation of α2PI levels with the incorporation of different α2PI forms into the plasma clot and different clot lysis parameters, bivariate (Pearson) correlation analyses were performed. For this analysis, non-normally distributed variables were naturally log-transformed to achieve normal distribution. An independent sample *t*-test was used to test differences in means of different clot lysis and clot structure parameters of plasma samples supplemented with recombinant α2PI. The level of significance was 95% (*p* < 0.05). Statistical analyses were performed using SPSS software (SPSS 28.0 for Macintosh, Chicago, IL, USA).

## 3. Results

### 3.1. Plasma Levels of Different Parameters in the Study Population

The role of NPB-α2PI in the regulation of fibrinolysis has not been previously investigated thoroughly. In this study, we intend to investigate how different α2PI forms influence clot lysis and clot structure. We randomly selected 80 plasmas from the healthy volunteers’ sample pool of our previous study [5] for the investigations. Characteristics of the study population are presented in Table 1. Each of the examined plasma parameters was in the reference range.

We found a robust correlation (r = 0.928, *p* < 0.001) between FXIII activity and antigen values (Supplementary Figure S1) and a strong correlation between α2PI activity and PB-α2PI antigen levels (r = 0.750, *p* < 0.001), as well as total-α2PI antigen levels (r = 0.691, *p* < 0.001). However, the correlation between α2PI activity and NPB-α2PI antigen levels was moderate (r = 0.325, *p* = 0.005) (Supplementary Figure S2).

### 3.2. Incorporation of α2PI Forms into the Plasma Clot

After clotting the plasma samples, we determined the clot-bound amounts of total-, PB-, and NPB-α2PI. Results are shown in Table 2. According to our calculation, 44.33 ± 6.3 percent of total-α2PI remained in the clot after centrifugation of the extruded serum, 57,8% of which was PB-α2PI. Compared to its original plasma level, a higher amount of NPB-α2PI was incorporated into the clot than PB-α2PI (58,07% vs. 37.62%, respectively). The incorporation of PB-α2PI showed a significant correlation with FXIII activity (r = 0.540, *p* < 0.001), fibrinogen level (r = 0.387, *p* < 0.001), and plasminogen level (r = 0.407, *p* < 0.001). The incorporation of NPB-α2PI did not show significant correlation with FXIII activity (r = 0.110, *p* = 0.331) and fibrinogen level (r = 0.086, *p* = 0.446), but correlated with plasminogen level (r = 0.322, *p* = 0.004).

### 3.3. Correlation of Different Parameters with Clot Lysis

The same plasma samples were also analyzed using a clot lysis assay, and clot formation and lysis were characterized based on CLT50, MaxAbs, and AUC values calculated during the analysis of turbidimetric curves. Correlations between parameters with a potential modifying effect on clot lysis (FXIII antigen and activity, fibrinogen, plasminogen, different forms of α2PI, and incorporated amount of α2PI forms) and parameters of clot lysis are shown in Table 3. The elevation of FXIII antigen and activity had a positive effect on MaxAbs and AUC, while they did not affect CLT50. The fibrinogen level only correlated with MaxAbs. The total-α2PI plasma level showed a statistically significant, positive correlation with all three lysis curve parameters. However, among the α2PI C terminal forms, this relationship was only observed in the case of NPB-α2PI. The plasma PB-α2PI/NPB-α2PI ratio showed a significant negative correlation with CLT50 and AUC (Supplementary Figure S3).

**Figure 1:**
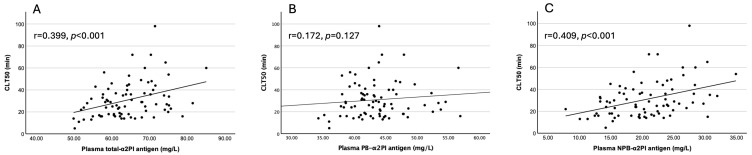
Correlation of plasma (**A**) total-α2PI, (**B**) PB-α2PI, and (**C**) NPB-α2PI levels with CLT50 (n = 80).

Interestingly, both plasma NPB-α2PI and incorporated NPB-α2PI antigen levels showed a statistically significant, positive correlation with CLT50, MaxAbs, and AUC, while for PB-α2PI, only the incorporated form correlated with MaxAbs (Figure 1A–C, Supplementary Figure S4).

### 3.4. Investigation of α2PI Incorporation into the Clot by Western Blot

**Figure 2:**
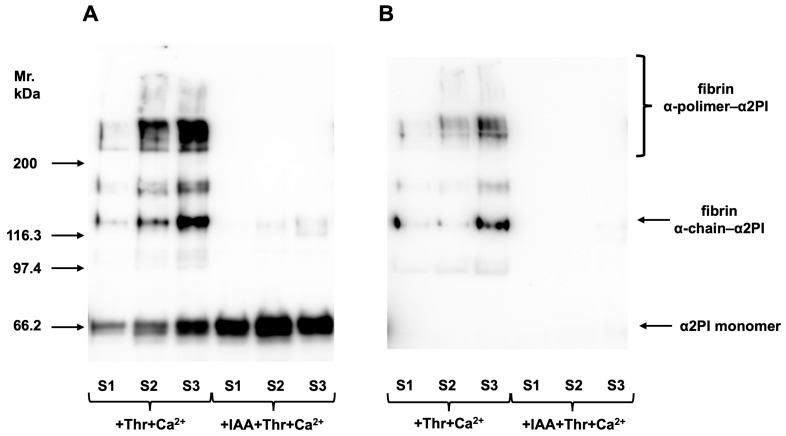
Three randomly selected control samples (S1–3) were clotted by adding thrombin and Ca^2+^, and after intensive washing, α2PI incorporated into the clot was investigated by Western blot. Polyclonal anti-α2PI antibody was used to detect all forms of bound α2PI (**A**), and a monoclonal anti-PB-α2PI specific antibody to detect the localization of the PB-α2PI form (**B**). Covalent binding of α2PI to fibrin α-chain and α-polymers can be seen after both staining, while the reaction corresponding to the monomer α2PI (noncovalently attached) can be seen only if the polyclonal antibody was used. In the presence of iodoacetamide, FXIII was inhibited. Therefore, the covalent binding did not occur, and only the monomer α2PI could be detected. Original images can be found in Supplementary Materials.

To investigate how α2PI forms incorporate into the clot, normal plasma samples (N = 3) were clotted and analyzed as described in Section 2.2.3. Polyclonal anti-α2PI antibody was used to detect all α2PI forms incorporated into the clot (Figure 2A). To localize the PB-α2PI form, a monoclonal antibody specific to PB-α2PI was used (Figure 2B). The α2PI crosslinked to the fibrin α-chain and α-polymers can be seen after both stainings, while the reaction corresponding to the monomer α2PI (noncovalently attached) can be seen only if the polyclonal antibody was used for detection. In the presence of IAA, FXIII was inhibited. Therefore, the covalent binding did not occur, and only the monomer α2PI could be detected. This noncovalently bound α2PI is supposedly the NPB-α2PI form, as it did not react with the PB-α2PI-specific antibody.

### 3.5. Investigation of Clot Lysis and Fibrin Structure Using Artificial Plasma Samples

**Figure 3:**
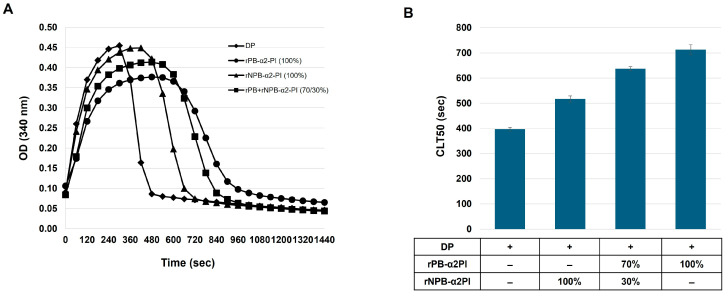
Effect of α2PI forms on clot lysis time. α2PI-deficient plasma was supplemented with recombinant PB- and/or NPB-α2PI; 100% corresponded to the estimated normal plasma concentration of α2PI (65 mg/L). (**A**) Clot lysis curves; (**B**) CLT50 values presented as mean ± SD values of 3 parallel measurements.

**Figure 4:**
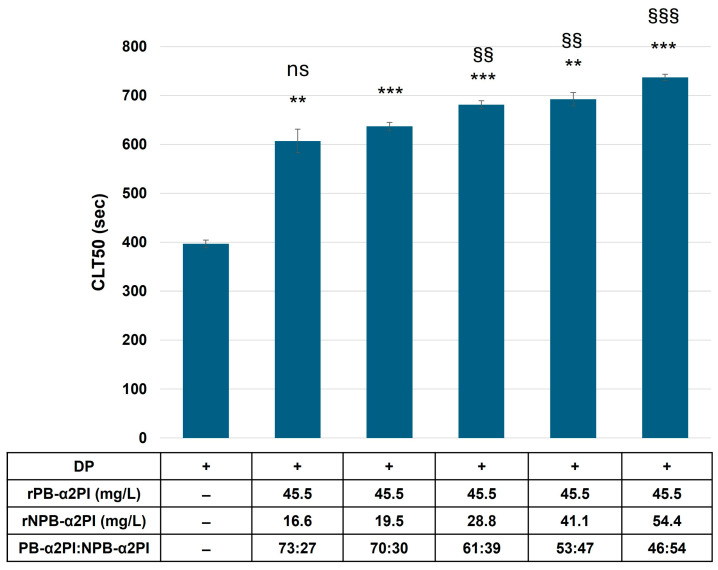
Effect of elevated NPB-α2PI on clot lysis time. α2PI-deficient plasma was substituted with an equal amount of recombinant PB-α2PI and an increasing amount of recombinant NPB-α2PI. CLT50 values are presented as mean ± SD values of 3 parallel measurements. ** *p* < 0.01 and *** *p* < 0.001 comparing to α2PI deficient plasma; ^ns^ non-significant, ^§§^ *p* < 0.01 and ^§§§^ *p* < 0.001 comparing to 70:30 PB-α2PI:NPB-α2PI ratio.

Our results showed that the PB-α2PI and NPB-α2PI levels correlated with the clot lysis parameters and both could be incorporated into the clot, albeit in different ways (Table 2, Figure 2). However, the heterogeneity of the N-terminal end of α2PI and variations in other influencing factors in real plasma samples make it difficult to detect true differences due to the C terminal truncation of α2PI. Therefore, next, we prepared plasma samples that differed only in the amount/ratio of the C-terminal forms of α2PI. α2PI-deficient plasma was supplemented with recombinant Met-PB- and/or Met-NPB-α2PI in different amounts, and a clot lysis assay was performed. Lysis time increased in the presence of both plasminogen-binding and non-binding forms of α2PI compared to the deficient plasma. Representative lysis curves are shown in Figure 3A. CLT50 was increased by 80% in the presence of 100% PB form (65 mg/L) and by 30% in the presence of 100% NPB-α2PI form (65 mg/L) compared to the α2PI-deficient plasma (Figure 3B). Next, we examined the effect of increasing NPB-α2PI on the lysis time while maintaining a constant amount of PB-α2PI form correspondingly close to the average normal plasma concentration (Figure 4). In parallel with the increase in the proportion of NPB-α2PI form, the lysis time increased, albeit slightly, but was statistically significantly (Figure 4). The highest amount of NPB-α2PI (when the PB-α2PI: NPB-α2PI ratio was 46:54%) caused a 21% elevation of CLT50 compared to the PB-α2PI: NPB-α2PI ratio of 73:27.

**Figure 5:**
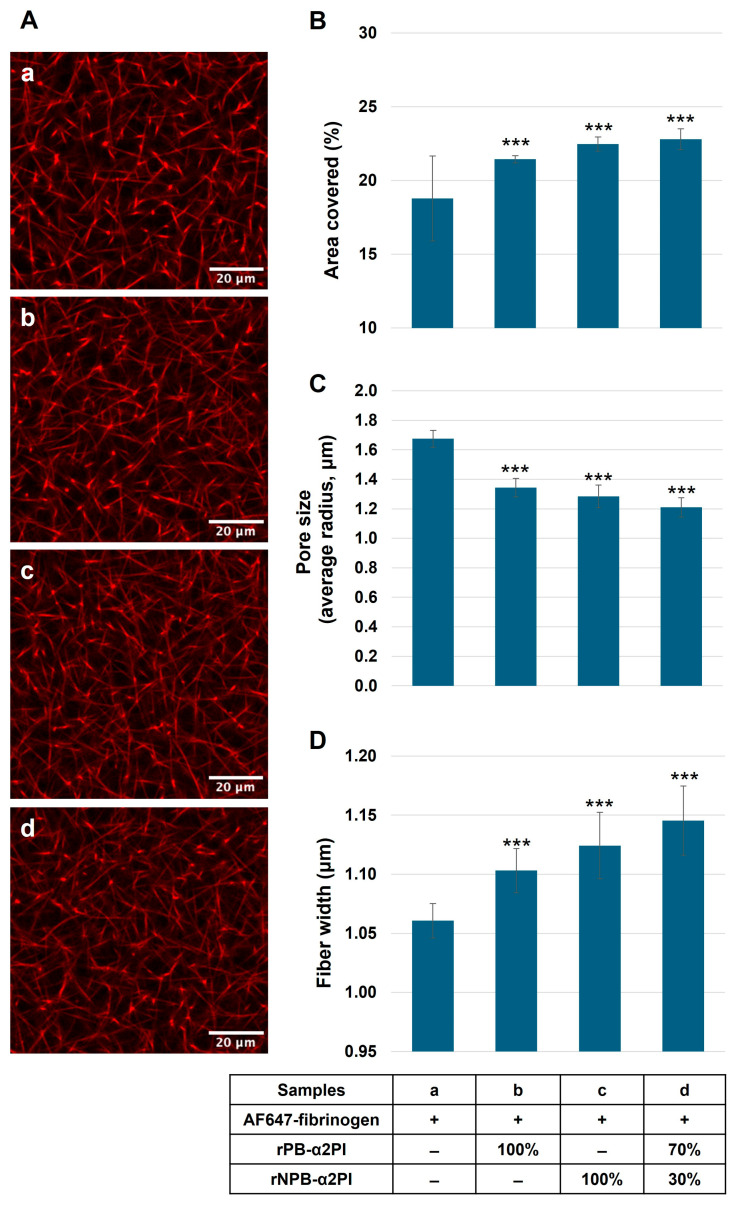
α2PI-deficient plasma supplemented with AF647-labelled fibrinogen, and different amounts of recombinant PB- and/or NPB-α2PI forms were clotted (samples a–d). And the washed clot was analyzed with confocal laser scanning microscopy (**A**). The percentage of area coverage was determined by Fiji software; pore size and the width of the fibrin fibers were calculated using a MATLAB (R2019a) GUI called CT-FIRE v3.0 beta (curvelet transform–fiber extraction). In the presence of PB and/or NPB forms, area coverage was significantly increased (**B**), pore size decreased (**C**), and thicker fibrin fibers evolved (**D**), therefore becoming a denser fibrin network in clots supplemented with rα2PI compared to the α2PI-deficient clot. 100% α2PI = 65 mg/L *** *p* < 0.001 comparing to α2PI deficient plasma, n = 22.

Next, we clotted α2PI-deficient plasma supplemented with AF647-labeled fibrinogen and recombinant Met-PB- and/or Met-NPB-α2PI in different amounts, and the structural architecture of the clot was analyzed with confocal laser scanning microscopy. Figure 5A demonstrates the effect of different ratios of α2PI forms on the fibrin structure in the clot. For quantitative analysis, we determined the percentage of area coverage, pore size, and width of the fibrin fibers (the original images (n = 22) from each channel are shown in Supplementary Figure S5a–d).

The covered area significantly increased in the presence of PB and/or NPB forms (Figure 5B). The pore size decreased (Figure 5C), and thicker fibrin fibers evolved (Figure 5D) in clots supplemented with rα2PI compared to the α2PI-deficient clot. The greatest difference compared to deficient plasma resulted when both forms were present in proportions like those described in normal plasma.

## 4. Discussion

**Figure 6:**
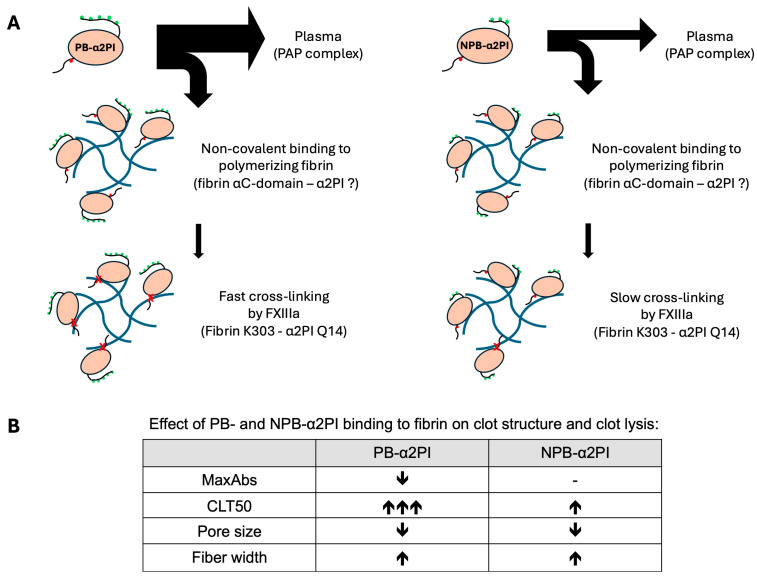
Putative mechanisms through which PB- and NPB-α2PI influence fibrin properties and clot lysis. (**A**) Binding of PB- and NPB-α2PI to fibrin: A higher amount of PB-α2PI is bound to the clot than NPB-α2PI. However, the amount of the fibrin-bound fraction relative to the original plasma concentration is lower for the PB variant compared to the NPB variant. (The thickness of the arrows reflects the concentration ratios detected in our experiments.). The site for the non-covalent interaction with fibrin is unknown in the α2PI molecule but is most likely different from the C-terminal part, as the interaction is Lys-independent [12]. When activated FXIII (FXIIIa) is present in sufficient quantities, PB-α2PI is rapidly cross-linked to fibrin and can no longer be detected in the clot in a non-covalently bound monomeric form. Conversely, NPB-α2PI is a less efficient substrate for FXIII, allowing it to be present in greater amounts in a non-covalently attached form within the clot. The green dots represent Lys residues in the C-terminal part of α2PI, which are important for binding to plasminogen. The red dot represents the FXIII cross-linking site (Q14), while red ‘x’ indicates covalent cross-linking. In the case of the NPB-α2PI variant, the last two Lys residues are absent, which significantly affects its interaction with plasmin, resulting in this variant being 10-times less potent as an inhibitor of plasmin [38]. (**B**) Effect of PB- and NPB-α2PI binding to fibrin on clot structure and clot lysis: PB-α2PI reduces the maximal clot density and significantly extends the clot lysis time in a concentration-dependent manner. In contrast, NPB-α2PI has no significant impact on clot density and only a moderate effect on lysis time. Both variants decrease pore size and increase the thickness of fibrin fibers.

α2PI undergoes proteolytic cleavages in the circulation, which have functional consequences. N-terminal cleavage by sFAP, which results in faster cross-linking to fibrin by FXIII, has been studied extensively. However, the extent and impact of C-terminal cleavage are less well-understood. C-terminal cleavage may affect the efficiency of plasmin inhibition, the degree and type of α2PI incorporation into the clot, and the structure of the clot. In this study, we provided new data on the effect of plasma levels and relative ratios of C terminally intact and truncated α2PI forms on their incorporation into clots, their influence on clot structure, and clot lysis. By measuring α2PI activity and antigen levels in parallel, we confirmed the previous assumption that activity measurement only shows a strong correlation with the concentration of the PB-α2PI form and is weakly influenced by the amount of the NPB form and is not influenced by the relative ratio of the two forms in the plasma (Supplementary Figure S2) [5,22]. Previously reported results on α2PI incorporation into plasma clots were largely influenced by the methodologies used. A Laurell assay showed a reduction of 18 ± 9% (n = 12) in total-α2PI antigen in serum compared to plasma, while the immediate plasmin inhibition test revealed a 35 ± 6% reduction in inhibition [19]. By measuring the α2PI activity, a 32.3% difference was reported between the plasma and serum levels of 65 blood donors [33]. By using an ELISA method to measure the total-α2PI in 6M urea solubilized clots, only a small fraction of α2PI, 1.35 ± 0.18 mg/L out of 83.2 ± 15.4 mg/L, was detected as a clot-bound fraction [34]. The method developed by Uitte de Willige et al. that measured the ratio of fluorescently labeled α2PI-specific antibody incorporation into the plasma clot amounted to 39 ± 4.9% crosslinking in clots prepared from citrated plasma from five healthy donors. They assumed that 90% of this amount was cross-linked, as they could only detect 3.9 ± 0.5% in the presence of FXIII inhibitor [35]. These results indicate that no method has been developed to date that can accurately detect the amount of cross-linked and non-covalently bound α2PI, as well as their ratio, in the clot. In our study, we determined the total-, PB-, and NPB-α2PI antigen levels parallel in plasma and serum samples of 80 healthy individuals, and incorporated amounts were calculated from the respective plasma and serum samples. The PB: NPB ratio in the plasma was 2.1 (1.75–2.57), which is in good agreement with the ratio of 2.2 (1.8–2.7) determined using the crossed immunoelectrophoresis technique with the addition of Lys-plasminogen to the first dimension gel [36]. Our results showed that 44.3 ± 6.3% of the total α2PI remained in the clot after serum removal, which is higher than the generally cited value of approximately 30%. The PB: NPB ratio in the clot was 1.37, which shows that a significant amount of NPB is bound to the clot, in a higher proportion than the PB form when compared to their original plasma levels. Western blot analysis of the extensively washed clots showed that FXIII crosslinks the PB form to fibrin, and this form cannot be detected in the clot in a non-covalently bound form. This confirms the previous results of Kluft et al. [19]. Non-crosslinked α2PI monomer was also visible on the blot when FXIII activity was inhibited. This form is presumably the NPB form, as the PB-specific antibody did not react with it (Figure 2). Consistent with this result, plasma FXIII levels showed a significant correlation with the incorporation of the PB form (Table 3) and not with the NPB form. It has previously been shown that the cross-linking of the PB form by FXIII is essential for preventing premature clot dissolution. Therefore, it was surprising to us that neither the plasma levels of FXIII nor the PB-form showed a significant correlation with the lysis time, but they did show a positive correlation with the MaxAbs. In contrast, both the plasma level and the incorporated amount of NPB form, which is a slow plasmin inhibitor, showed a significant correlation with all three clot lysis parameters. To further investigate this effect, we supplemented α2PI-deficient plasma with full-length and/or C terminally truncated recombinant α2PI and examined the effect of supplementation on clot lysis and clot structure. The NPB form, although to a much lesser extent than the PB form, was able to extend the lysis time, and if increasing amounts of the NPB form were added to a constant amount of PB form corresponding to approximately normal plasma levels, further prolongation was observed. Both forms of α2PI affected the clot structure, increasing the thickness of fibrin fibers and reducing the pore size. Because the two forms bind to fibrin by different mechanisms, we were able to detect the greatest effect when both forms were present in proportions like those in normal plasma, rather than when only one form was present, albeit at a 100% normal plasma concentration. Mechanisms that determine fibrin structure are not yet fully understood. However, numerous studies have reported alterations in fibrin structure associated with various thrombotic conditions. In several studies, reduced pore size, formation of thinner fibrin fibers, and reduced permeability have been linked to decreased fibrinolytic capacity and increased risk of thrombosis. Conversely, other studies report the formation of thicker fibrin fibers along with increased lysis time and reduced permeability [37]. These latter findings support our results. However, in our case, the impact of clot structural changes on fibrinolysis caused by the binding of α2PI to fibrin cannot be separated from direct plasmin inhibition. It can be assumed that both mechanisms contribute to the reduction of the overall fibrinolytic capacity. Figure 6 summarizes the putative mechanisms through which α2PI C-terminal variants influence fibrin properties.

Summarizing the previous literature data in line with the results of our study, it seems that the cross-linking of the fast-acting PB-α2PI form to fibrin by FXIII is crucial for preventing early fibrin mesh degradation. However, the incorporation of the PB form is limited to approximately 38% of the amount in plasma. The NPB form is non-covalently incorporated into the clot independently of FXIII activity and, although a slow plasmin inhibitor, can further enhance the inhibition of fibrinolysis, but it is not sufficient on its own to prevent early fibrinolysis.

This study should be interpreted in the context of its limitations. The study group is rather young, with a mean age of 33.2 ± 13.4 years, which could influence the generalizability of the results. The lack of reaction with the PB-α2PI-specific antibody is only indirect evidence that the PB variant is not present in the clot due to non-covalent binding to fibrin. It cannot be excluded that non-covalent binding to fibrin renders the epitope of the antibody used inaccessible.

## 5. Conclusions

In conclusion, in our study, we found that NPB-α2PI can bind non-covalently to fibrin, and this binding contributes to changes in clot structure and inhibition of fibrinolysis. Since most previous clinical studies only examined α2PI activity, which does not reflect the plasma levels of NPB-α2PI, little is known about the amount of NPB-α2PI in different diseases. Therefore, further studies are needed to investigate the extent and effect of C terminal truncation in different pathological conditions.

## Figures and Tables

**Table 1:** Characteristics of the study population (n = 80).

Parameters	Value	Reference Range
Age (years)	33.2 ± 13.4	na
Gender (male%)	36.3	na
Plasma levels of		
FXIII activity (%)	109.70 ± 26.77	69–143
FXIII-A_2_B_2_ ag (mg/L)	22.36 ± 5.07	14–28
Fibrinogen (g/L)	3.42 ± 0.55	1.5–4.0
Plasminogen (%)	105.0 (95.5–118.5)	75–150
Total-α2PI ag (mg/L)	64.49 ± 7.56	48–85
PB-α2PI ag (mg/L)	43.12 (40.46–45.99)	-
PB-α2PI % (% of total-α2PI)	68.03 ± 6.14	-
NPB-α2PI ag (mg/L)	20.83 ± 5.53	-
PB-α2PI/NPB-α2PI ratio	2.1 (1.75–2.57)	-

Values with normal and non-normal distributions are expressed as mean ± SD and median (IQR). na: non-applicable.

**Table 2:** α2PI amounts incorporated into the plasma clot and clot lysis parameters (n = 80).

Parameters	Mean ± SD/Median (IQR)
α2PI incorporation	
Incorporated total-α2PI ag (mg/L)	28.58 ± 5.6
Incorporated total-α2PI ag (% of plasma total-α2PI)	44.33 ± 6.3
Incorporated PB-α2PI ag (mg/L)	16.51 ± 3.8
Incorporated PB-α2PI (% of plasma PB-α2PI)	37.62 ± 6.5
Incorporated NPB-α2PI ag (mg/L)	12.07 ± 4.2
Incorporated NPB-α2PI (% of plasma NPB-α2PI)	58.07 ± 13.5
Clot lysis	
CLT50 (min)	28.0 (18.0–38.5)
MaxAbs (OD)	1.26 ± 0.16
AUC (mODxmin)	22.60 ± 8.15

Values with normal and non-normal distributions are expressed as mean ± SD and median (IQR).

**Table 3:** Correlation of FXIII activity, FXIII antigen, fibrinogen, plasminogen, different forms of α2PI in plasma, and clot-bound α2PI forms with clot lysis curve parameters (CLT50, MaxAbs, and AUC) (n = 80).

Parameters	CLT50	MaxAbs	AUC
FXIII activity (%)	0.127 (0.261)	**0.438** (<0.001)	**0.332** (0.003)
FXIII-A_2_B_2_ ag (mg/L)	0.050 (0.662)	**0.374** (<0.001)	**0.270** (0.015)
Fibrinogen (g/L)	0.039 (0.733)	**0.518** (<0.001)	0.120 (0.291)
Plasminogen activity (%)	0.087 (0.451)	**0.434 (<0.001)**	0.183 (0.112)
Plasma total-α2PI ag (mg/L)	**0.399** (<0.001)	**0.249** (0.026)	**0.349** (0.002)
Plasma PB-α2PI ag (mg/L)	0.172 (0.127)	0.125 (0.270)	0.151 (0.182)
Plasma NPB-α2PI ag (mg/L)	**0.409** (<0.001)	**0.231** (0.040)	**0.353** (0.001)
Plasma PB-α2PI/NPB-α2PI ratio	**−0.327** (0.003)	−0.172 (0.127)	**−0.294** (0.008)
Incorporated total-α2PI ag (mg/L)	**0.317** (0.004)	**0.458** (<0.001)	**0.396** (<0.001)
Incorporated PB-α2PI ag (mg/L)	0.136 (0.229)	**0.295** (0.008)	0.208 (0.064)
Incorporated NPB-α2PI ag (mg/L)	**0.298** (0.007)	**0.344** (0.002)	**0.339** (0.002)

Values are expressed as Pearson’s r (significance *p*). Statistically significant correlations are highlighted in bold characters.

## Data Availability

The raw data supporting the conclusions will be made available by the authors, without undue reservation.

## Supplementary Materials

The following supporting information can be downloaded at https://www.mdpi.com/article/10.3390/biom15081127/s1: Supplementary Figure S1. Correlation of plasma FXIII activity with FXIII antigen levels; Supplementary Figure S2. Correlation of plasma α2PI activity with total-, PB-, NPB-α2PI antigen levels, and percentage of NPB-α2PI in the plasma; Supplementary Figure S3. Correlation of plasma PB-α2PI/NPB-α2PI ratio with MaxAbs, CLT50, and AUC; Supplementary Figure S4. Correlation of incorporated total-, PB-, and NPB-α2PI antigen levels with MaxAbs, CLT50, and AUC; Confocal laser scanning microscopy images of the in vitro plasma clots.

## Abbreviations

The following abbreviations are used in this manuscript:

α2PI	Alpha2-plasmin inhibitor, alpha2-antiplasmin
APCE	Antiplasmin cleaving enzyme
AUC	Area under the curve
CLT50	50% clot lysis time
DP	Deficient plasma
ELISA	Enzyme-linked immunosorbent assay
FXIII	Blood coagulation factor XIII
HRP	Horseradish peroxidase
IAA	Iodoacetamide
NPB-α2PI	Plasminogen non-binding alpha2-plasmin inhibitor
PAP-complex	Plasmin-α2-antiplasmin complex
PB-α2PI	Plasminogen-binding alpha2-plasmin inhibitor
sFAP	Soluble fibroblast activation protein
TAFI	Thrombin-activatable fibrinolysis inhibitor
t-PA	Tissue-type plasminogen activator
