# Supplementary material for: Effect of Alpha2-Plasmin Inhibitor C-Terminal Heterogeneity on Clot Lysis and Clot Structure
Source: Biomolecules. 2025 Aug 5;15(8):1127. doi: 10.3390/biom15081127 (PMC12384537; doi:10.3390/biom15081127)
Supplement: Supplementary file 1 [file biomolecules-15-01127-s001.zip › Supplementary Files/Supplementary figures.pdf]

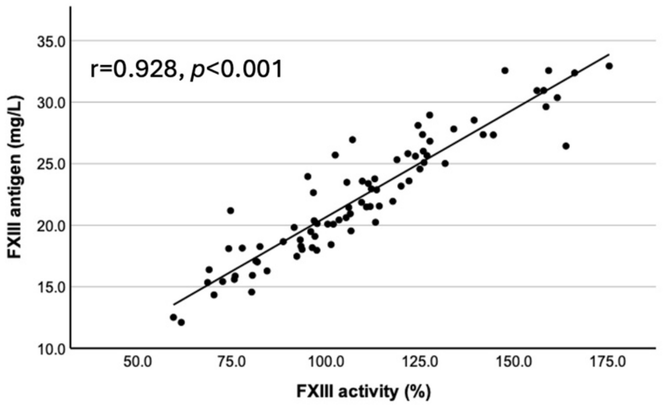

Supplementary Figure S1. Correlation of plasma FXIII activity with FXIII antigen levels (n=80).

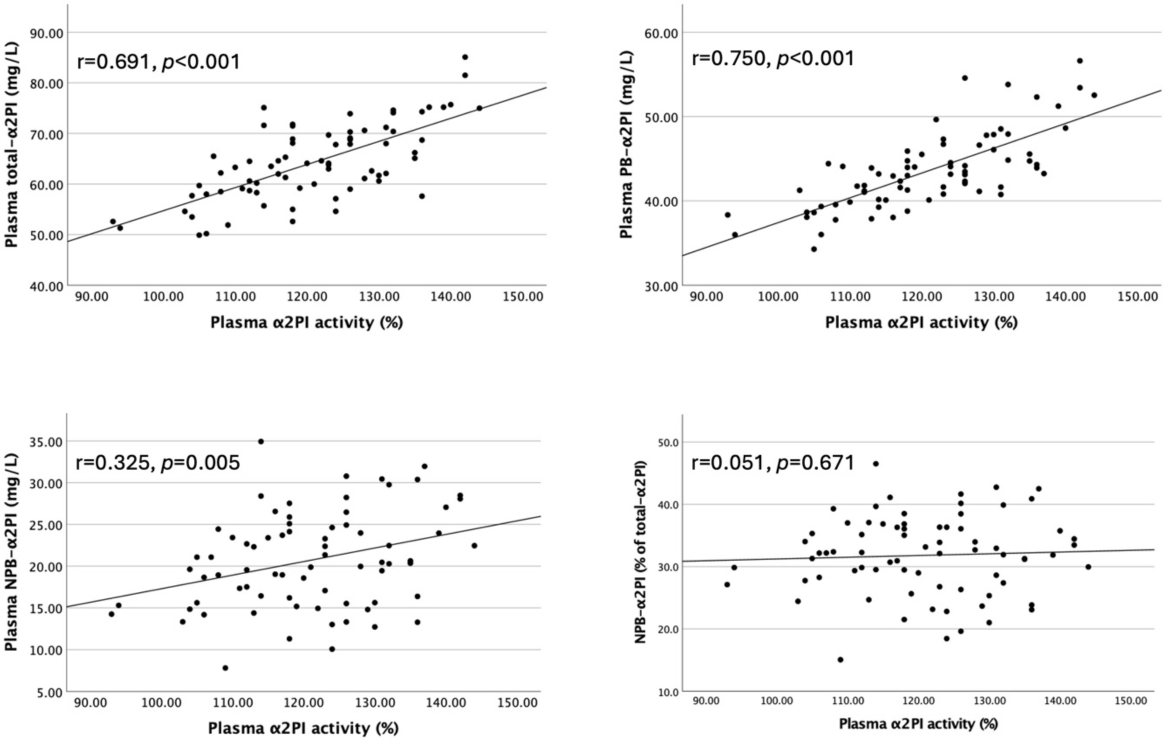

Supplementary Figure S2. Correlation of plasma α2PI activity with total-, PB-, NPB-α2PI antigen levels, and percentage of NPB-α2PI in the plasma (n=80).

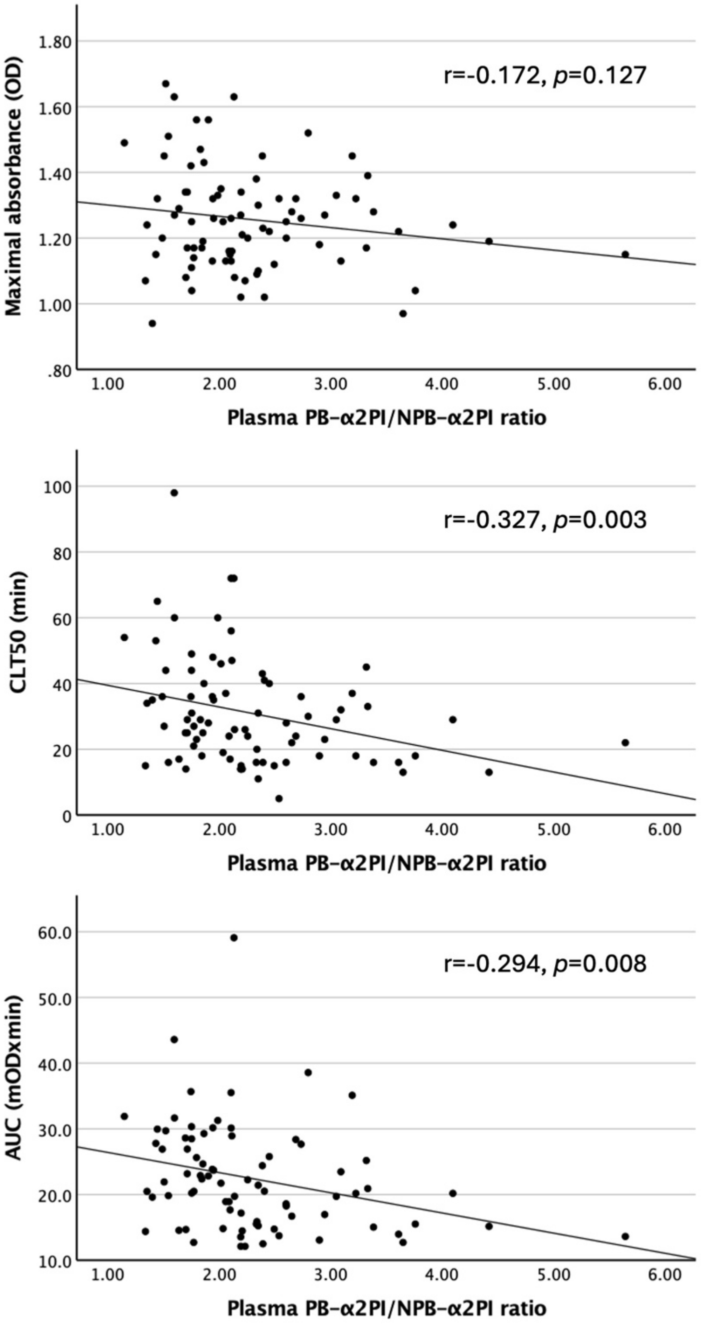

Supplementary Figure S3. Correlation of plasma PB-α2PI/NPB-α2PI ratio with MaxAbs, CLT50, and AUC (n=80).

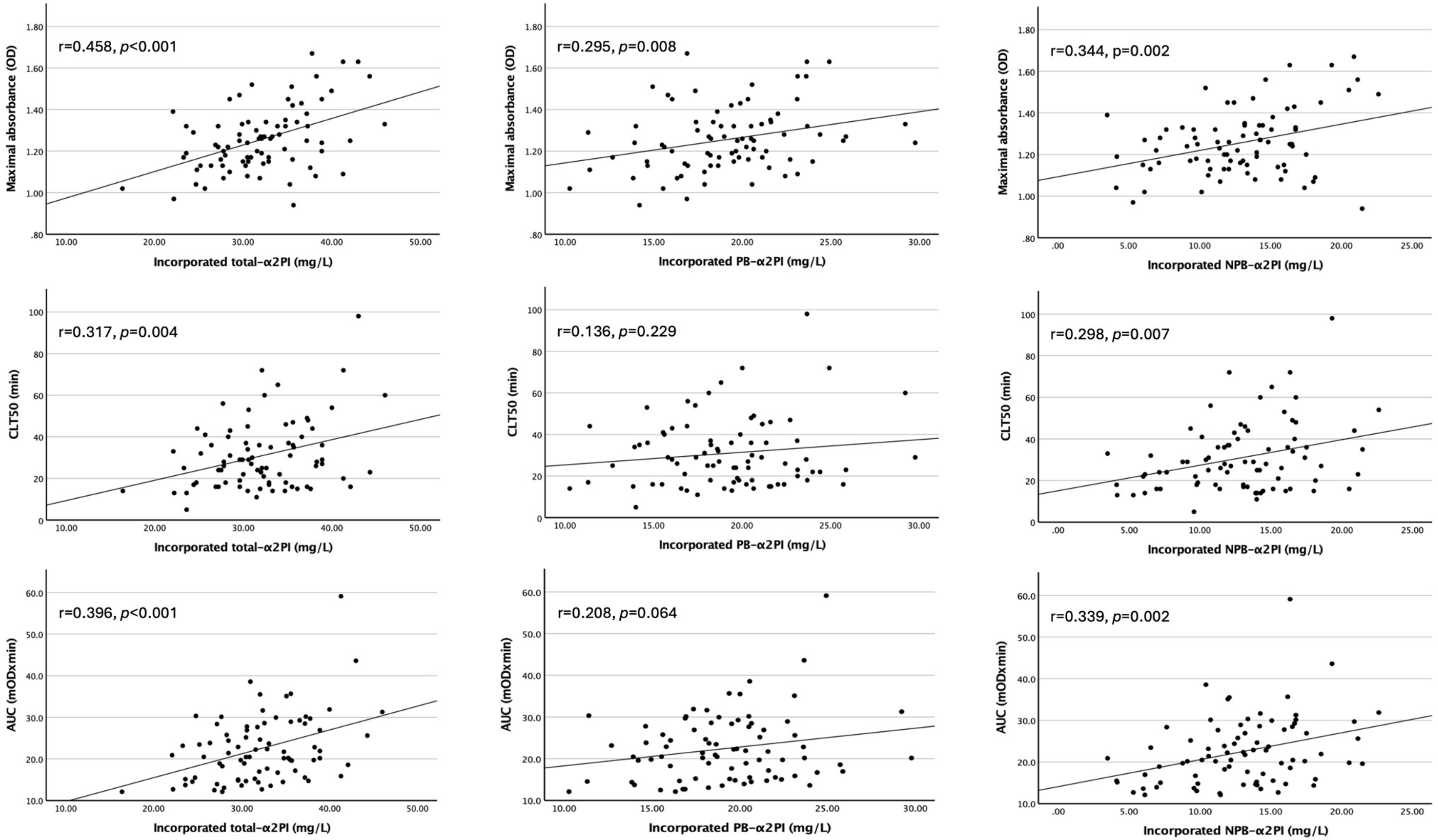

Supplementary Figure S4. Correlation of incorporated total-, PB-, and NPB-α2PI antigen levels with MaxAbs, CLT50, and AUC (n=80).

Confocal laser scanning images of the *in vitro* plasma clots. α2PI-deficient plasma samples supplemented with AF647-labelled fibrinogen and different amounts of recombinant PB- and/or NPB-α2PI forms were clotted in channels of an Ibidi μ-Slide VI, and the washed clots were analysed with confocal laser scanning microscopy. Twenty-two images (1024x1024 pixels) from each channel (samples a-d) were recorded in the same positions in a Z-stack.
